# ERBB activation modulates sensitivity to MEK1/2 inhibition in a subset of driver-negative melanoma

**DOI:** 10.18632/oncotarget.4255

**Published:** 2015-06-13

**Authors:** Katherine E. Hutchinson, Douglas B. Johnson, Adam S. Johnson, Violeta Sanchez, Maria Kuba, Pengcheng Lu, Xi Chen, Mark C. Kelley, Qingguo Wang, Zhongming Zhao, Mark Kris, Michael F. Berger, Jeffrey A. Sosman, William Pao

**Affiliations:** ^1^ Department of Cancer Biology, Vanderbilt University Medical Center, Nashville, Tennessee, USA; ^2^ Department of Medicine/Division of Hematology-Oncology, Vanderbilt University Medical Center, Nashville, Tennessee, USA; ^3^ Department of Pathology, Microbiology & Immunology, Vanderbilt University Medical Center, Nashville, Tennessee, USA; ^4^ Department of Biostatistics, Vanderbilt University Medical Center, Nashville, Tennessee, USA; ^5^ Department of Surgery, Division of Surgical Oncology and Endocrine Surgery, Vanderbilt University Medical Center, Nashville, Tennessee, USA; ^6^ Department of Biomedical Informatics, Vanderbilt University Medical Center, Nashville, Tennessee, USA; ^7^ Department of Medicine, Memorial Sloan-Kettering Cancer Center, New York, USA; ^8^ Human Oncology and Pathogenesis Program, Memorial Sloan-Kettering Cancer Center, New York, USA; ^9^ Department of Pathology, Memorial Sloan-Kettering Cancer Center, New York, USA; ^10^ Currently an employee of Roche Pharma Research and Early Development, Basel, Switzerland

**Keywords:** melanoma, ERBB, DUSP4, trametinib, afatinib

## Abstract

Melanomas are characterized by activating “driver” mutations in BRAF, NRAS, KIT, GNAQ, and GNA11. Resultant mitogen-activated protein kinase (MAPK) pathway signaling makes some melanomas susceptible to BRAF (BRAF V600 mutations), MEK1/2 (BRAF V600, L597, fusions; NRAS mutations), or other kinase inhibitors (KIT), respectively. Among driver-negative (“pan-negative”) patients, an unexplained heterogeneity of response to MEK1/2 inhibitors has been observed. Analysis of 16 pan-negative melanoma cell lines revealed that 8 (50%; termed Class I) are sensitive to the MEK1/2 inhibitor, trametinib, similar to BRAF V600E melanomas. A second set (termed Class II) display reduced trametinib sensitivity, paradoxical activation of MEK1/2 and basal activation of ERBBs 1, 2, and 3 (4 lines, 25%). In 3 of these lines, PI3K/AKT and MAPK pathway signaling is abrogated using the ERBB inhibitor, afatinib, and proliferation is even further reduced upon the addition of trametinib. A potential mechanism of ERBB activation in Class II melanomas is minimal expression of the ERK1/2 phosphatase, DUSP4, as ectopic restoration of DUSP4 attenuated ERBB signaling through potential modulation of the ERBB ligand, amphiregulin (AREG). Consistent with these data, immunohistochemical analysis of patient melanomas revealed a trend towards lower overall DUSP4 expression in pan-negative versus BRAF- and NRAS-mutant tumors. This study is the first to demonstrate that differential ERBB activity in pan-negative melanoma may modulate sensitivity to clinically-available MEK1/2 inhibitors and provides rationale for the use of ERBB inhibitors, potentially in combination with MEK1/2 inhibitors, in subsets of this disease.

## INTRODUCTION

Malignant melanoma is comprised of molecular subsets characterized by constitutively activating “driver” mutations in the serine-threonine kinase BRAF (codon V600), the GTPase NRAS (G12, G13, and Q61), the receptor tyrosine kinase KIT (W557, V559, L576, K642, and D816), and the Gα GTPases GNAQ (Q209) and GNA11 (Q209) [1–5]. Importantly, all of these mutations have been shown to activate the mitogen-activated protein kinase (MAPK) signaling pathway. Notably, BRAF V600E and KIT kinase domain mutations are associated with high sensitivity to targeted BRAF (vemurafenib, dabrafenib) or KIT (imatinib, nilotinib) small molecule inhibitors, respectively [6–12]. In addition to BRAF-specific inhibition with vemurafenib and dabrafenib, the MEK1/2 inhibitor trametinib is also approved for the treatment of metastatic or unresectable BRAF V600-mutant melanoma [13–16]. The optimum treatment for other subsets, including NRAS-, GNAQ- or GNA11-mutant melanomas, remains to be determined.

Despite the exciting advances in targeted treatment for melanoma, up to one-third of tumors express none of these driver mutations, herein called “pan-negative”. Because they have no identifiable drug target, treatment options for these patients are extremely limited. Chemotherapy may be utilized but has limited efficacy and no clear survival benefit. “Targeted” immunotherapies such as ipilimumab (anti-cytotoxic T-lymphocyte antigen-4/anti-CTLA-4) and anti-programmed death-1/programmed death 1-ligand 1 (anti-PD-1/PD-L1) monoclonal antibodies, are emerging as effective treatment for both driver-positive and -negative melanomas, but efficacy is not uniform and many patients fail to respond [17–20]. More recently, in melanomas previously considered pan-negative for common driver mutations, we identified non-V600 BRAF mutations at codons L597 and K601 [21] and BRAF fusions [22]. Importantly, both alterations activate the MAPK pathway and the induced signaling confers sensitivity to MEK1/2 inhibition. As a result of these studies, MEK1/2 inhibitors are being evaluated for use in the BRAF non-V600-mutant and BRAF fusion subsets through an ongoing, multicenter Phase II clinical trial (NCT02296112). Taken together, these data suggest that constitutive activation of the MAPK pathway is a critical factor in the pathogenesis of most melanomas.

Current evidence suggests that many pan-negative melanoma cell lines are sensitive to MEK1/2 inhibitors without a known molecular basis [23, 24]. Based on these observations, and because the majority of currently known driver mutations in melanoma result in MAPK pathway activation, an open question is whether all pan-negative melanomas could be treated with MEK1/2 inhibitors. Here, we investigated sensitivity to MEK1/2 inhibition in 16 pan-negative melanoma cell lines and found that differences in ERBB activation and DUSP4 expression may modulate responses. In the future, these studies may lead to novel clinical trials involving pharmacological inhibition of ERBB family members in combination with established MEK1/2 inhibitors in otherwise untreatable, pan-negative melanoma.

## RESULTS

### Pan-negative melanomas display differential sensitivity to MEK1/2 inhibition

**Figure 1:**
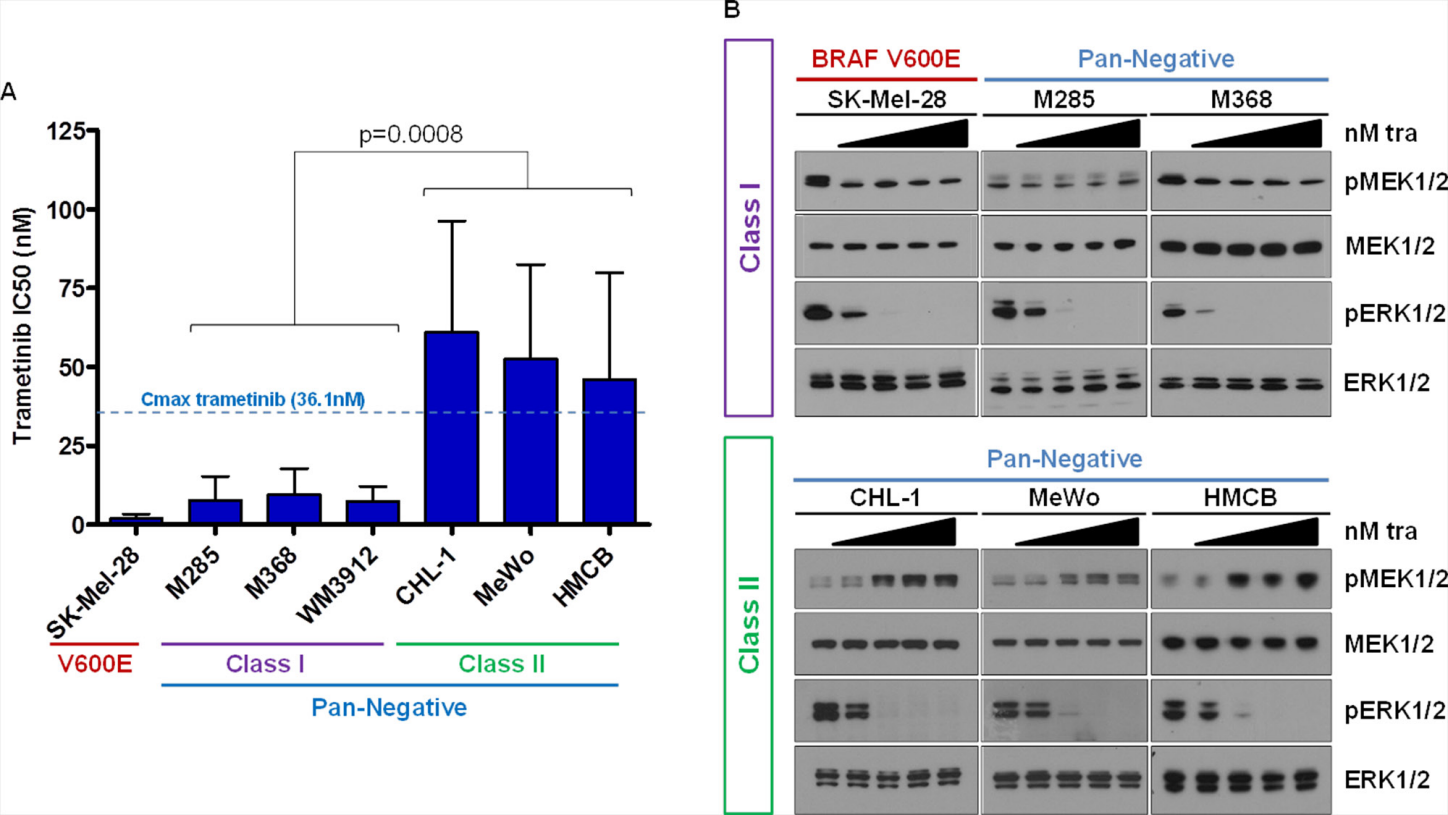
Pan-Negative Melanomas Display Differential Sensitivity to MEK1/2 Inhibition **A.** IC50s for a panel of 6 pan-negative melanoma lines and one BRAF V600E line to trametinib were determined by standard growth inhibition assays with increasing concentrations of drug. Class I cells display IC50's well below the trametinib C_max_ and similar to that of the V600-mutant line; Class II lines exhibit IC50s above the trametinib C_max_. **B.** Paradoxical activation of MEK1/2 is observed in Class II cells upon trametinib treatment. nM, nanomolar; tra, trametinib; p, phosphorylated. The *p*-value was calculated using Student's *T*-test, assuming unequal variance.

At Vanderbilt, patient melanomas are routinely screened for well-established and targetable driver point mutations in BRAF (codon V600), NRAS (G12, G13, and Q61), KIT (W557, V559, L576, K642, and D816), GNAQ (Q209) and GNA11 (Q209) with a multiplex-PCR and capillary electrophoresis-based assay termed SNaPshot (Supplementary Table S1) [25]. Herein, we used SNaPshot to identify melanoma cell lines that were pan-negative by this assay (Supplementary Table S2). To determine potential differences in the sensitivity of pan-negative melanomas to MEK1/2 inhibition, we treated an initial collection of six SNaPshot pan-negative melanoma cell lines (Supplementary Table S3) with the clinically-available MEK1/2 inhibitor, trametinib, and calculated the average IC50 for each in comparison to a well-described BRAF V600E-mutant melanoma line, SK-Mel-28. Interestingly, we observed two distinct responses. Three pan-negative lines were highly sensitive to trametinib with IC50's relatively similar to SK-Mel-28 (BRAF V600-mutant) and well below the C_max_ for trametinib (36.1 nM, [26]). The other three pan-negative lines exhibited IC50's at or above the C_max_ of trametinib (Figure 1A). When we investigated MAPK pathway signaling in these six cell lines following treatment with trametinib, the lines less sensitive to trametinib displayed paradoxical activation of MEK1/2 (Figure 1B and Supplementary Figure S1). To distinguish between the two groups, we herein termed the two MEK1/2-inhibitor response groups as Class I and Class II, respectively.

Paradoxical MEK1/2 activation following MEK1/2 inhibitor administration has been reported previously in the setting of a Ras mutation [27, 28], a BRAF V600 mutation and concurrent MEK1 mutation [29], or BRAF amplification and MEK2 mutation [30, 31] (Supplementary Table S4). Targeted next-generation sequencing (Vanderbilt Cancer Panel for MiSeq and MSKCC IMPACT Assay, refer to Supplementary Materials and Methods and Supplementary Tables S5 and S6, and see [32]) did not detect any RAS G12, G13, or Q61 codon mutations (NRAS, KRAS or HRAS) or MEK1/2 (a.k.a. MAP2K1, MAP2K2) mutations in the six pan-negative melanoma cell lines. A non-canonical BRAF N581Y alteration was detected by IMPACT analysis in WM3912. This alteration is predicted to induce modest BRAF kinase activity, but is poorly characterized [33, 34] (Supplementary Table S6). Although RAS activity in both classes was slightly less than that of an NRAS-mutant cell line, we observed no significant difference in RAS activity between the two pan-negative classes (Supplementary Figure S2).

### Class II pan-negative melanoma lines exhibit active ERBB receptors

**Figure 2:**
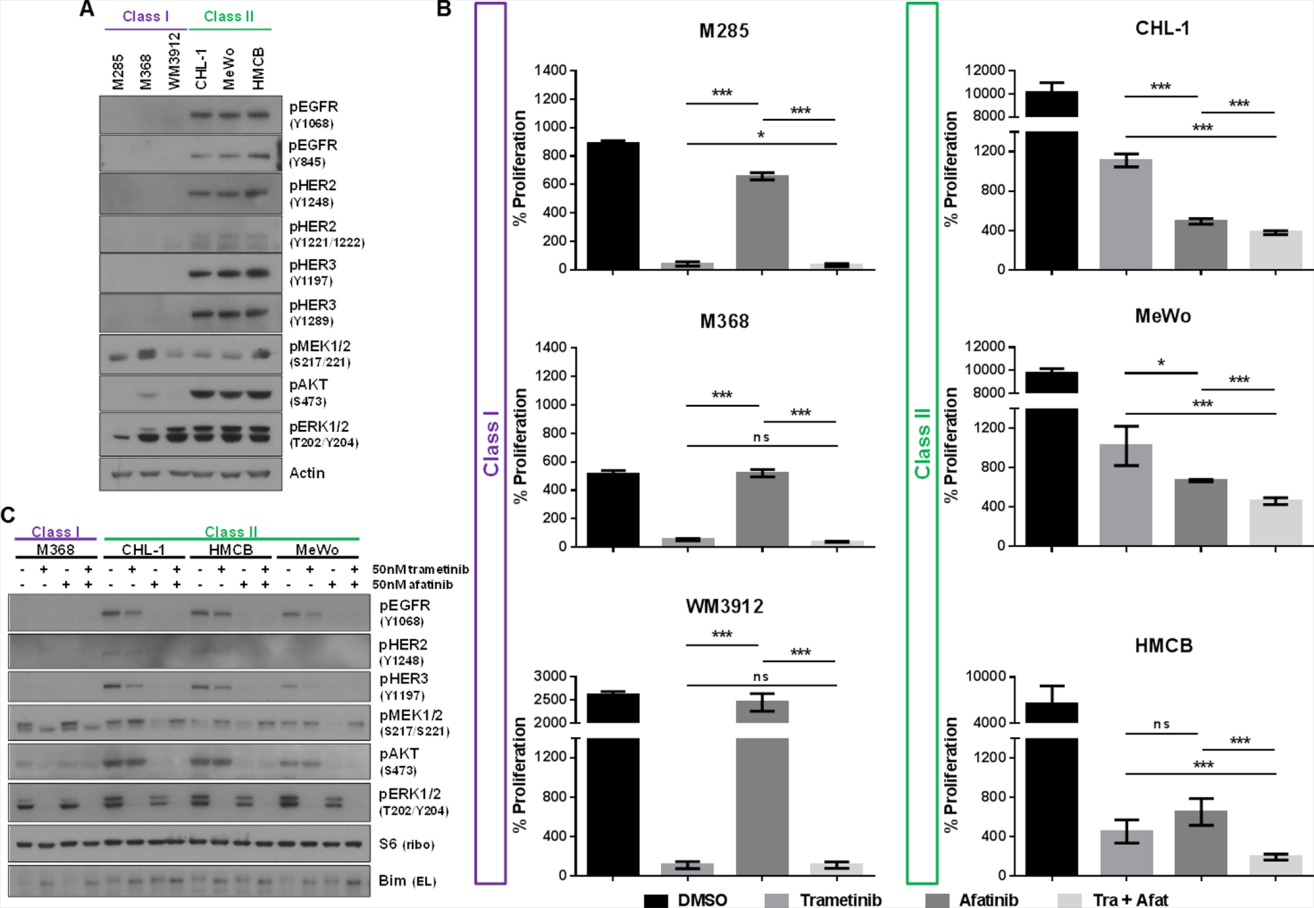
Class II Pan-Negative Melanoma Lines Exhibit Active ERBB Receptors and are Sensitive to ERBB Kinase Inhibition **A.** Immunoblotting analysis reveals that only Class II pan-negative cells express endogenously phosphorylated EGFR, HER2 and HER3, in addition to phospho-AKT. All cells were cultured in the presence of serum. **B.** After 6 days of proliferation in vehicle (DMSO), 50 nM trametinib, 50 nM afatinib, or the combination, only the Class II lines are sensitive to single-agent afatinib. Additionally, the combination is more effective at inhibiting proliferation of Class II cells than either single agent. **C.** Immunoblotting analysis of Class I and II cells following treatment with DMSO, 50 nM trametinib, 50 nM afatinib or the combination shows that phosphorylation of EGFR, HER2, HER3 and AKT is diminished upon afatinib treatment, but only the combination abolishes signaling of both AKT and ERK1/2 in these lines. nM, nanomolar; p, phosphorylated; tra, trametinib; afat, afatinib. *p*-values for Figure 2B were calculated using Student's *T*-test, assuming unequal variance, where ***p < 0.01, *p < 0.05 and ns = not significant.

A previous study of cancer cells not specific to melanoma reported paradoxical activation of MEK1/2 upon MEK1/2 inhibition in a BRAF-/NRAS-wild-type setting, citing that signaling in these cells is regulated by receptor tyrosine kinases (RTKs) [35] (Supplementary Table S4). We investigated the RTK status of Class I and II lines by commercial phosphorylated RTK array and immunoblotting analysis. The phospho-RTK array indicated that all lines exhibited some degree of low-level EGFR activity (Supplementary Figure S3A), but only Class II lines displayed activation of HER2/ERBB2 and HER3/ERBB3 receptors. Subsequent immunoblotting analysis confirmed that only Class II lines exhibited endogenous levels of phosphorylated EGFR, HER2 and HER3 receptors (Figure 2A). There were no stark differences in expression of total EGFR, HER2 and HER3 between Class I and II cells, though Class II cells may harbor slightly increased levels of these proteins (Supplementary Figure 3B). ERBB-phosphorylated Class II lines also displayed elevated levels of phosphorylated AKT, suggesting that Class II cells may be dependent not only on MAPK pathway signaling, but also the PI3K/AKT pathway.

### Class II pan-negative melanoma lines are sensitive to EGFR small-molecule inhibition

Because Class II lines demonstrated active EGFR, HER2 and HER3, we next investigated their potential sensitivity to the ERBB-targeting small molecule inhibitors, afatinib (irreversible, inhibits EGFR > HER2 > HER3) and lapatinib (reversible, inhibits HER2 > EGFR). Cell viability and proliferation analyses confirmed that only Class II lines were sensitive to afatinib and lapatinib, whereas Class I cells were resistant to either agent (afatinib, Figure 2B, Supplementary Figures S4A, S4B; lapatinib, data not shown). Additionally, treatment with single-agent afatinib ablated AKT phosphorylation in Class II lines (Figure 2C).

To determine whether Class II cells would be more sensitive to combined inhibition of the ERBBs and MEK1/2, we administered both afatinib and trametinib to the Class II cells. The combination had some effect on cell viability (Supplementary Figures S4A, S4B), and enhanced inhibition of proliferation in Class II cells, while no added effect was observed in Class I cell proliferation (Figure 2B). Furthermore, combined inhibition of ERBBs and MEK1/2 attenuated both AKT and ERK1/2 phosphorylation, causing a slight increase in levels of the pro-apoptotic protein, BIM, in Class II cells (Figure 2C, Supplementray Figure S4c).

### ERBB and AKT activation status may predict sensitivity to MEK1/2 inhibition

**Figure 3:**
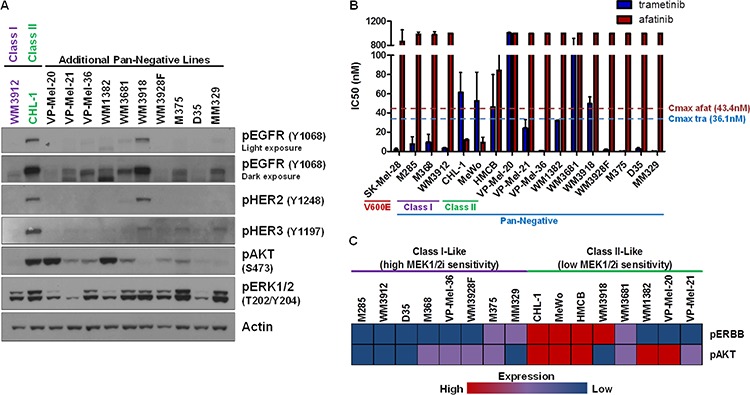
ERBB and AKT Activation Status May Predict Sensitivity to MEK1/2 Inhibition **A.** Immunoblotting analysis of 10 additional pan-negative melanoma lines reveals that phosphorylated ERBB and AKT status is variable in the pan-negative subset, with one additional line (WM3918) exhibiting obvious ERBB activity. **B.** Summary of growth inhibition assay-derived IC50's for the 16 pan-negative melanoma lines (including Class I and II lines) and a BRAF V600-mutant line (SK-Mel-28, for comparison) to afatinib and trametinib. **C.** A rough clustering analysis of the expression of phospho-EGFR/HER2/HER3 (pERBB) and phospho-AKT by immunoblot in (A) reveals differences between Class I-like and Class II-like pan-negative melanomas. p, phosphorylated; tra, trametinib; afat, afatinib; nM, nanomolar.

To determine the frequency of ERBB activation in pan-negative melanomas, we expanded our cohort to 10 additional SNaPshot pan-negative lines (16 total) from various institutions (Supplementary Table S3). Interrogation of the phospho-ERBB status of these 10 lines by immunoblot analysis revealed one additional line (WM3918) with clearly active EGFR, HER2 and HER3 (Figure 3A). None of the additional lines were sensitive to afatinib (Figure 3B). Five of the additional lines (VP-Mel-36, WM3928F, M375, D35, MM329) displayed a Class I phenotype in that they were highly sensitive to trametinib (IC50 << trametinib C_max_) but resistant to afatinib, indicating that 8 of 16 (50%) of these pan-negative melanoma cell lines were Class I-like. A rough clustering of the cell lines analyzing expression of phosphorylated ERBBs 1, 2, and 3 and phosphorylated AKT as observed by immunoblot analysis across the 16 lines (Figure 3C) revealed that Class I-like lines with high sensitivity to MEK1/2 inhibition displayed very little to no phosphorylated ERBBs or AKT. Among Class II-like lines, the only lines sensitive to afatinib were CHL-1, HMCB, and MeWo, which, in addition to ERBB phosphorylation, also exhibited activated AKT. In contrast, while WM3918 cells expressed high phospho-EGFR, they were not responsive to afatinib and lacked phosphorylated AKT. Further, no EGFR, HER2 or HER3 mutations were identified in this cell line by the MSKCC IMPACT assay that would lead to afatinib resistance (Supplementary Table S6). The other Class II-like lines (WM1382, VP-Mel-20, VP-Mel-21) exhibited no phospho-ERBBs but had high or intermediate activation of AKT. Notably, two lines (VP-Mel-20 and WM3681) were susceptible to neither ERBB nor MEK1/2 inhibition. Clearly, there may be sub-classes within the Class I, Class II designations that are influenced by other, as yet undetermined signaling pathways.

We should note that the IMPACT assay detected non-canonical BRAF alterations in VP-Mel-20 (G469R), VP-Mel-21 (N581I), and as reported above, in WM3912 (N581Y) (Supplementary Table S6). These mutations are reported to confer MAPK pathway activity but to a lesser extent than a BRAF V600 alteration [3, 33, 34]. Previously, it has been shown that a cell line harboring both BRAF G469A and BRAF L584F was sensitive to the BRAF V600-mutant inhibitor, vemurafenib, but insensitive to trametinib [36], which is consistent with our findings. Limited data are available regarding the sensitivity of tumors harboring the N581 alteration to such inhibitors. Our results suggest this particular mutation may have no bearing on whether a melanoma is Class I or II, as one N581-mutant cell line was represented in each class (Figure 3C).

In summary, 8 of 16 (50%) pan-negative cell lines displayed a Class I phenotype. Of the lines with decreased sensitivity to MEK1/2 inhibition (Class II phenotype), ERBB activity was observed in 4 of these 16 lines (25%), and 3 (18.8% of total) were sensitive to afatinib. Because ~30% of melanomas are currently considered pan-negative, one could extrapolate that ~6% of all melanomas are Class II-like, however, future studies with clinical specimens will provide more robust evidence.

### Lack of DUSP4 is a potential mechanism for ERBB activation in class II melanomas

**Figure 4:**
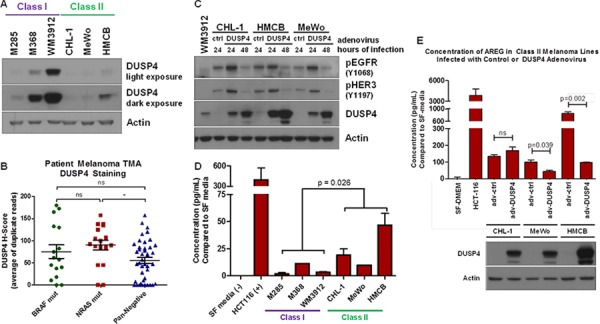
Lack of DUSP4 is a Potential Mechanism for ERBB Activation in Class II Melanomas **A.** Immunoblot analysis of DUSP4 expression in Class I and II pan-negative melanomas reveals that Class II melanomas express little or no DUSP4 compared with Class I melanomas. **B.** Plot of H-scores for immunohistochemistry against DUSP4 in 15 BRAF-mutant, 17 NRAS-mutant and 42 pan-negative melanomas on a patient tissue microarray (TMA) reveals a wide distribution of DUSP4 expression in all three subsets, but a trend toward lower overall DUSP4 expression in the pan-negative group. **C.** Restoring DUSP4 expression in Class II melanoma lines by adenovirus infection leads to a reduction in active EGFR and HER3. **D.** By protein array, amphiregulin (AREG) levels are higher in Class II melanomas compared with Class I melanomas, which was confirmed by ELISA, shown here. Serum-free media and conditioned media from HCT116 cells were used as negative and positive controls, respectively. The *p*-value was calculated by Student's *T*-test assuming unequal variances. **E.** Restoring DUSP4 in Class II cells decreases the expression of AREG in MeWo and HMCB Class II cell lines. The *p*-value was calculated by Student's *T*-test assuming equal variances. adv, adenovirus; ctrl, control; SF media, serum-free media, p, phosphorylated.

RTK-activated cancers, unlike BRAF V600E-activated cancers, may harbor lower levels of the ERK1/2 phosphatase, DUSP4 [35]. To determine whether this was the case in Class II pan-negative melanomas, we analyzed the six original Class I & II cells by immunoblot analysis and observed DUSP4 expression existed primarily in Class I cells, whereas Class II cells harbored little to no DUSP4 expression (Figure 4A). Immunohistochemical analysis of BRAF-mutant, NRAS-mutant and pan-negative patient melanomas revealed wide heterogeneity of DUSP4 expression in each subtype. Consistent with our *in vitro* data and Class I/Class II subtyping, a large number of pan-negative tumors exhibited no DUSP4 expression (Figure 4B). Although differences in EGFR expression were difficult to show in Class I and II cell lines, Class II lines potentially harbored greater levels of HER2 and HER3 than Class I lines. RNA sequencing analysis of DUSP4, EGFR, HER2 and HER3 expression in pan-negative melanomas genotyped through The Cancer Genome Atlas (TCGA) revealed an inverse relationship between DUSP4 and EGFR expression (*p*-value = 0.03978) (Supplementary Figure S5). Interestingly, when DUSP4 was restored in Class II cells, EGFR and HER3 activation was diminished by 48 hours (Figure 4C).

Once activated, ERK1/2 can serve as a transcriptional co-regulator of various proteins, including ERBB ligands [37]. Since DUSP4 negatively regulates ERK1/2 activity, ERK1/2-mediated transcription of ERBB ligands may be altered between Class I and II cell lines. Therefore, we examined whether Class II cells secreted higher levels of ERBB ligands compared to Class I cells. Analysis of conditioned media for four of seven ERBB ligands (epidermal growth factor, EGF; heparin-binding epidermal growth factor, HB-EGF; heregulin / neuregulin β1, HRG / NRGβ1; amphiregulin, AREG) by protein array revealed potentially higher levels of HB-EGF and AREG in Class II cells (Supplementary Figure S6A, S6B). Subsequent enzyme-linked immunosorbent assays (ELISAs) for HB-EGF and AREG confirmed upregulation of AREG without clear differences in HB-EGF expression (Figure 4D, Supplementary Figure S6C). In addition to suppressing ERBB activity, DUSP4 restoration in Class II cells decreased AREG expression in two of the three Class II cell lines (Figure 4E). These data suggest that a potential mechanism of constitutive ERBB activation in Class II cells may be associated with lower levels of DUSP4, which allows for ERK1/2-mediated transcription of ERBB ligands.

## DISCUSSION

The identification of MAPK-pathway activating driver mutations in BRAF, NRAS, KIT, GNAQ and GNA11 in melanoma has revolutionized the treatment of this disease beyond standard chemotherapy to include targeted, small-molecule inhibitors such as vemurafenib, dabrafenib, imatinib, nilotinib and trametinib. Unfortunately, only two-thirds of patients harbor these drivers, leaving the remaining one-third of “pan-negative” patients with no targeted treatment option. Since melanoma is widely considered to be dependent on MAPK pathway signaling for its growth and survival, we sought to determine if there are subsets of pan-negative melanoma that display differential sensitivities to MAPK pathway inhibition in order to ultimately identify novel therapeutic options for patients.

**Figure 5:**
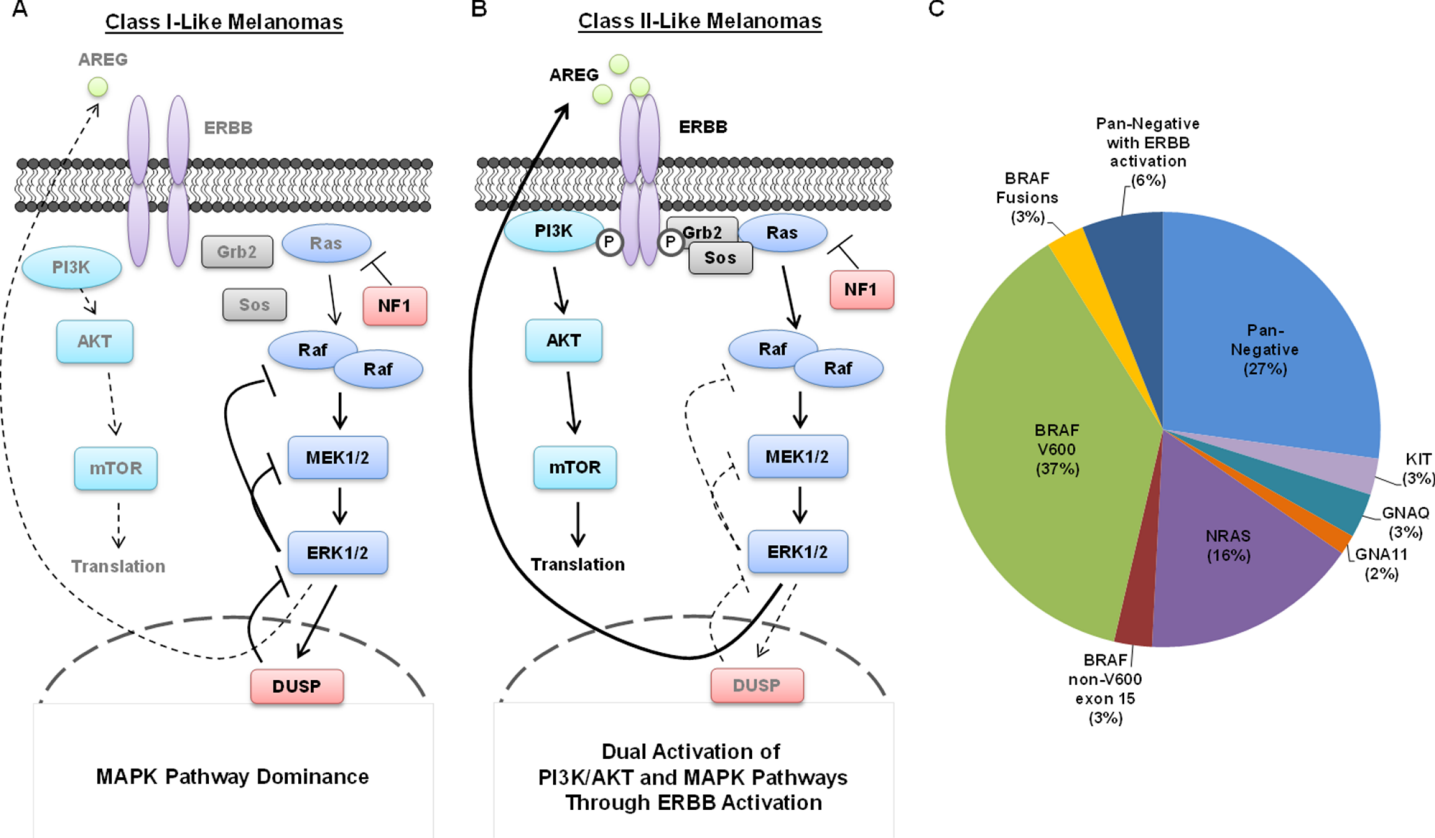
Summary of Class I and Class II Pan-Negative Melanomas **A.** Class I pan-negative melanomas are primarily driven by the MAPK pathway and express generally robust levels of the ERK1/2 phosphatase, DUSP4, but have no endogenously active ERBB receptors. Class I melanomas behave similarly to BRAF V600-mutant melanomas in that they are highly sensitive to MEK1/2 inhibition. **B.** Class II pan-negative melanomas are activated by ERBB receptors, which activate both MAPK and PI3K/AKT signaling, making them less susceptible to MEK1/2 inhibition than Class I melanomas, but more susceptible to combined inhibition of ERBBs and MEK1/2. A potential mechanism for the ERBB activity in Class II's is the relative lack of DUSP4 expression, presumably allowing ERK1/2-mediated transcription of ERBB ligands such as amphiregulin (AREG). **C.** This pie chart displays the breakdown of driver events that sustain melanoma. Our study has shown that 4 pan-negative melanomas display ERBB activation (25%), but only 3 of those (18.8%) were sensitive to ERBB inhibition which can be extrapolated to ~6% of all melanomas.

We ascertained that there are two possible MEK1/2-inhibitor response “classes” within pan-negative melanomas (Table 1, Figure 5): Class I pan-negative responders behave like BRAF V600-mutant cells in that they are highly sensitive to MEK1/2 inhibition and downregulation of phosphorylated MEK1/2 and ERK1/2 is observed. Class II pan-negative responders, by contrast, are less sensitive to MEK1/2 inhibition and display paradoxical activation of MEK1/2 upon treatment. These pan-negative melanomas did not harbor mutations in MEK1 or 2 [29–31], nor did they display increased RAS activity [27, 28], both of which are implicated in previous citings of paradoxical MEK1/2 activation upon MEK1/2 inhibition, specifically in the BRAF V600-mutant setting (Supplementary Table S4). Another study, however, suggested that tumors driven by receptor tyrosine kinases (RTKs) would exhibit paradoxical MEK1/2 activation after MEK1/2 inhibition [35]. Interestingly, only the Class II pan-negative responders displayed basal activation of EGFR, HER2 and HER3, associated with phosphorylation of downstream AKT (Figure 2). Importantly, we show that Class II cells respond well to the EGFR > HER3 > HER2 inhibitor, afatinib, and combining afatinib with trametinib elicits even greater effects on proliferation and signaling than either single agent (Figure 2B, 2C). We also reveal that a potential mechanism of heightened ERBB activity in Class II pan-negative melanomas is the relative lack of DUSP4 expression, a negative regulator of ERK1/2 (Figure 4). By restoring expression of DUSP4 in these cells, both amphiregulin (AREG) expression and ERBB activity was diminished (Figure 4C, 4E). Consistent with our *in vitro* data, analysis of tissue microarrays and RNA sequencing expression data from The Cancer Genome Atlas (TCGA) revealed a wide distribution of DUSP4 expression in pan-negative samples (Figure 4B) and an inverse relationship between DUSP4 and EGFR expression (Supplementary Figure S5).

**Table 1:** Summary of BRAF V600-mutant and Pan-Negative (PN) Class I and II Phenotypes

Trametinib Response Class		BRAF V600E	PN Class I	PN Class II
**ImmunoBlot**	**pMEK1/2**	↓	↓	↑
	**pERK1/2**	↓	↓	↓
**Growth Inhibition Assay**	**vemurafenib**	S	R	R
	**trametinib**	S	S	I
**ERBB Activation**		No	No	Yes
**ERBBi Sensitivity**		No	No	Yes
**DUSP Expression**		Yes	Yes	No
**% of Pan-Negatives**		n/a	50%	18.8%
**% of all melanomas**		~40%	~15%	~6%

↓, decrease in activity; ↑, increase in activity; S, sensitive; R, resistant; I, intermediate sensitivity; ERBBi, ERBB inhibitor

In addition to identifying ERBB activation in some pan-negative melanomas, interrogation of a larger panel of pan-negative melanoma cell lines revealed the potential for non-ERBB-dependent mechanisms of MEK1/2 inhibitor resistance as well (Figure 3). For example, although WM3918 cells were Class II-like in their limited response to trametinib, unlike the other Class II lines with ERBB activity, WM3918 did not respond to afatinib. Since these cells did not display phosphorylated AKT and did not harbor ERBB mutations known to cause resistance to this agent, they may be dependent on other, as yet unidentified, signaling pathways in addition to the MAPK pathway. Furthermore, the pan-negative lines VP-Mel-20 and WM3681 were resistant to both MEK1/2 and ERBB inhibition. Studies are ongoing to determine the pathway(s) and mechanism(s) that distinguish these melanomas from our proposed Class I / Class II subtypes. One possible explanation for differences in MEK1/2 inhibitor response may relate to MITF and/or AXL expression levels. In the BRAF V600-mutant setting, low AXL/high MITF expression was shown to correlate well with MEK1/2 inhibitor sensitivity [38, 39]. To our knowledge, differences in this signaling axis have not been specifically interrogated in the setting of pan-negative melanoma.

Activation of receptor tyrosine kinases (RTKs) and specifically, ERBBs, through mutation [40–46] or amplification/overexpression [47–51] represent major hallmarks of cancers such as lung and breast. Unlike these cancer types, RTK/ERBB amplification/overexpression is not considered an intrinsic characteristic of melanoma. Furthermore, previous pre-clinical studies investigating the potential for ERBB inhibition in this disease never correlated genotype (BRAF-mutant, NRAS-mutant, or pan-negative) to ERBB activation and/or response to ERBB-directed therapy as we have [52, 53]. ERBB inhibitors, potentially in combination with RAF inhibitors, may prove useful in the setting of acquired resistance to RAF inhibitors in BRAF V600-mutant melanoma [54–58], but acquired resistance to first-line therapy was not a focus of our study.

To our knowledge, only one clinical trial exists that evaluated ERBB inhibition in melanoma. This phase II trial investigated the efficacy of gefitinib, an EGFR tyrosine kinase inhibitor, in a population of 48 patients with melanoma, unselected for genotype [59]. Although the median progression-free and overall survival figures were 1.4 and 9.7 months, respectively, there were two partial responders (4%) with a medium duration of response of 10.9 months. For comparison, the median progression-free survival of BRAF V600-mutant patients on vemurafenib is 6.8 months [12]. It is possible that these two “exceptional responders” had Class II-like melanoma, harboring activated EGFR. Unfortunately, we were unable to obtain tumor specimens from these two responders to further investigate their ERBB and/or DUSP4 status. Notably, however, this figure supports our data, suggesting that perhaps 6% of melanomas are pan-negative with ERBB activation (Figure 5C). Again, these theories would benefit from future analysis of clinical specimens.

Our study helps further define a potential role for ERBB activity in pan-negative melanoma and how that activity might modulate therapeutic responses, specifically to MEK1/2 inhibitors. Our observation of endogenous ERBB activity and resultant sensitivity to ERBB inhibition in 3 of 4 Class II-like pan-negative lines suggests that ERBB inhibitors may be efficacious in this disease, potentially in combination with MEK1/2 inhibitors.

In line with these endeavors, it will be important to determine the appropriate clinical test by which to identify Class II patients. Because this phenotype is not represented by a single driver mutation (point mutation, insertion, deletion, etc), the assay must detect differences in protein levels between tumor and normal tissues. Next-generation whole-genome or RNA sequencing analyses are certainly useful, but cannot provide protein expression or phosphorylation information. Immunohistochemical (IHC) methods, both traditional and quantitative [60], may be more suitable. Because an inverse relationship between total EGFR and DUSP4 expression was observed in TCGA RNA sequencing data (Supplementary Figure S5), samples could be analyzed for total levels of EGFR and DUSP4. Mass cytometry (a.k.a. CyTOF) alternatively has several advantages over traditional IHC methods. Not only does mass cytometry allow for highly quantitative, single-cell analyses of either a few or several targets, but it is also useful for phospho-protein analyses [61]. Furthermore, mass cytometry has the ability to analyze live, single-cell suspensions as well as image whole fixed tissues. Ideally, CyTOF could be used to compare the levels of phosphorylated ERBBs, phosphorylated AKT, and total DUSP4 levels in tumor and matched normal samples from each patient.

In summary, we have identified a subset of pan-negative melanoma with reduced sensitivity to MEK1/2 inhibition that is mediated by an axis involving ERBB activation/DUSP4 expression. Interrogation of a large number of pan-negative melanoma cell lines for ERBB activity and sensitivity to trametinib or afatinib revealed that this Class II phenotype potentially represents 18.8% of pan-negative melanomas, or ~6% of all melanomas. As stated above, this number is supported by a phase II clinical trial evaluating gefitinib in melanoma in which 4% of the population exhibited responses [59]. Furthermore, these data suggest that ERBB inhibition may be a therapeutic option for a subset of patients whose melanomas are considered pan-negative.

## MATERIALS AND METHODS

### Cell lines

The sources and culturing conditions of all 16 pan-negative melanoma lines are listed in Supplementary Table S3. Fetal bovine serum (FBS) was heat-inactivated (Atlanta Biologicals) and the penicillin-streptomycin solution was at a final concentration of 100 U/mL penicillin and 100 μg/mL streptomycin (Mediatech). RPMI-1640 (Mediatech #MT10040 CV), DMEM (Gibco/Life Technologies #11965). SK-Mel-28 was provided through MTA with Christine Pratilas (Memorial Sloan-Kettering Cancer Center) to Kimberly Dahlman (Vanderbilt) and cultured in DMEM + 10% FBS + 1% pen/strep. Wistar Institute cell lines were cultured in a solution of 4 parts MCDB-153 media (Sigma, #M7403) to 1 part Leibovitz's L-15 medium (Gibco/Life Technologies, #11415–064) and also containing 2% FBS, 1% pen/strep, 5 ug/mL bovine insulin (Sigma # I5500), and 1.68 mM calcium chloride (VWR #97062–586). The HCT116 colorectal cancer cell line was kindly provided by Robert Coffey (Vanderbilt) and cultured in RPMI + 10% FBS + 1% pen/strep. VP-Mel cell lines were derived from patient melanomas; patients gave consent for the use of their tissue under VICC-MEL0287. All cells were tested in-house for mycoplasma contamination and confirmed to be negative. Additionally, all melanoma cell lines were subjected to the Vanderbilt SNaPshot assay for melanoma (Supplementary Table S2), which has been described previously [25] and recently updated to include BRAF L597Q (c.1790T > A), R (c.1790T > G), and S (c.1789_1790 CT > TC) and BRAF K601E (c.1801A > G), to confirm genotype (Supplementary Table S1).

### Antibodies

Phospho-EGFR (Y1068) was from Abcam (ab40815). Total-EGFR was from BD Biosciences (#610017). Actin was from Sigma (#A2066). The following antibodies were from Cell Signaling: Phospho-antibodies against EGFR Y845 (#2231), HER2 Y1248 (#2247), HER2 Y1221/1222 (#2243), HER3 Y1197 (#4561), HER3 Y1289 (#4791), MEK1/2 S217/221 (#9154), AKT S473 (#4060), ERK1/2 T202/Y204 (#9101); total antibodies against DUSP4 (#5149), Bim (#2819), HER2 (#2242), HER3 (#4754), MEK1/2 (#9126), AKT (#9272), ERK1/2 (#9102).

### Drugs/Adenovirus/siRNA

Trametinib (GSK1120212) was from Chemietek. Afatinib was synthesized by the Organic Synthesis Core Facility at Memorial Sloan-Kettering Cancer Center under the direction of Ouathek Ouerfelli. DUSP4 adenovirus was described previously [62] and kindly provided by Justin Balko (Vanderbilt). Pooled small interfering RNA's against HER3 (SMARTpool: ON-TARGETplus ERBB3 siRNA) and a pooled scrambled control (On-TARGET plus non-targeting siRNA pool) were from Dharmacon (L-003127–00-0005 and D-001810–10-05, respectively).

### Growth inhibition assays

Cells were seeded at 3,000 cells per well of a 96-well plate. Following 4- or 5- day treatment with DMSO or increasing doses of drug in sextuplicate, Cell Titer Blue reagent (Promega) was added to each well and fluorescence was measured as per manufacturer's instructions on a BioTek microplate reader.

### Immunoblotting

All cells were lysed on ice using standard RIPA buffer (50 mM Tris-HCl, pH 7.5; 150 mM NaCl; 1% IGEPAL/NP-40 substitute; 0.1% SDS) and supplemented with protease and phosphatase inhibitors (Roche Complete Mini Protease Inhibitor cocktail tablet, EDTA-free, used as per manufacturer's instructions; 40 mM sodium fluoride; 1 mM sodium orthovanadate; 1 μM okadaic acid). Cells were not allowed to reach >85–90% confluence before harvesting. Lysates were quantified by Bradford assay and subjected to SDS-PAGE on 4–12% Bis-Tris gels (Invitrogen/Life Technologies). Following transfer to PVDF membranes, immunoblot analysis was performed using antibodies against the indicated targets. Membranes were incubated in chemiluminescent reagents (Perkin Elmer) and exposed to film for signal detection.

### DUSP4 adenovirus infection

Cells were plated evenly into 6-cm dishes in serum-containing media. The following day, media was replaced with a solution of 1.5 μL of a control GFP adenovirus or DUSP4-containing adenovirus, 500 μL of serum-free media, and 50 uL of 1 M HEPES solution (25 mM final concentration). Plates were rocked every 15 minutes for 1 hr. Finally, 1.5 mL serum-containing media was added to each plate (total volume ~2 mL). 24 hr after adenovirus infection, cells were treated with DMSO or 50 nM trametinib for 24 hr. Cells were harvested for immunoblotting or serum-free conditioned media was harvested for ELISAs as described in other sections.

### Enzyme-linked immunosorbent assays (ELISAs)

One day following even seeding into 6-cm dishes, serum-containing culture media was replaced with serum-free culture media. After 48 hr, the conditioned media was harvested, spun at 4°C to pellet loose cells/debris, and used as per manufacturer's instructions for the HB-EGF and AREG ELISAs (Abcam, ab100531 and ab99975, respectively).

### Tissue microarray (TMA) immunohistochemistry for DUSP4

The melanoma TMA was created using formalin-fixed, paraffin-embedded tissues from 17 BRAF-mutant (14 V600E, 3 V600K), 17 NRAS (8 Q61R, 4 Q61K, 2 Q61L, 1 Q61H, 1 G13V, 1 G12C) and 49 pan-negative melanoma patients seen at Vanderbilt. All patient tissues were reviewed for ≥50% tumor content and assessed using the Vanderbilt melanoma SNaPshot assay [25] to confirm mutation status. Immunohistochemistry was performed for DUSP4 (Cell Signaling #5149) as described previously [62] according to the following parameters: antigen retrieval using citrate buffer, pH 6.0 (decloaking chamber); dilution of 1:400; overnight incubation at 4°C; and the Envision Visualization System from Dako. Tumor regions stained for nuclear DUSP4 were assessed using a four value intensity scale (0 to 3) and percentage extent (0 to 100%). The H-score was calculated by summing the products of both parameters (range 0–300). The Kruskal-Wallis rank sum test was applied to the TMA data and *post hoc* analyses were performed for pair-wise comparisons among the three groups (BRAF-mutant, NRAS-mutant or pan-negative).

## SUPPLEMENTARY METHODS






