# Supplementary material for: ERBB activation modulates sensitivity to MEK1/2 inhibition in a subset of driver-negative melanoma
Source: Oncotarget. 2015 Jun 13;6(26):22348–60. doi: 10.18632/oncotarget.4255 (PMC4673168; doi:10.18632/oncotarget.4255)
Supplement: Supplementary file 1 [file oncotarget-06-22348-s001.pdf]

SUPPLEMENTARY METHODS

Ras-GTP assay

To detect active Ras (Ras-GTP) levels, we used the Active Ras Pull-Down and Detection Kit from ThermoScientific (#16117). Cells were plated evenly into 10-cm dishes with serum-containing media. 24 hr later, media was aspirated and replaced with serum-containing or serum-free media for 12 hr. Lysates were harvested and resuspended in 300 uL of the kit's Lysis/Binding/Wash buffer supplemented with protease and phosphatase inhibitors as described in the main methods ("Immunoblotting"). Lysates were quantified by BCA Assay (ThermoScientific #23225). For the Ras-GTP pulldown, lysates were normalized to 500 µg in 500 µL Lysis/Binding/Wash buffer. For whole-cell lysate controls, lysates were normalized to 30 µg in 20 µL buffer + Laemmli dye. *In vitro* GTPγS and GDP treatment, and the active Ras precipitation were performed as per manufacturer's instructions. Both the precipitations and whole-cell lysates were subjected to SDS-PAGE and transfer to PVDF as described in the main methods ("Immunoblotting"). Active (pulldown) and total (WCL) Ras were detected using the anti-Ras antibody provided with the kit as per manufacturer's instructions.

Phosphorylated receptor tyrosine kinase (RTK) Arrays

To assess phosphorylated RTK status, we used R&D Systems' Proteome Profiler Human Phospho-RTK Array kit (ARY001B). Cells were plated evenly into 6-cm dishes and cultured in the presence of serum. After 48 hr, lysates were harvested in the kit's Lysis Buffer 17, which was supplemented with protease and phosphatase inhibitors as described above. Lysates were quantified by Bradford assay and normalized to 400 µg per 1.5 mL lysate + Lysis Buffer 17 solution. Phospho-RTK array membranes were blocked as per manufacturer's instructions and the lysate solutions added to the membranes. All remaining steps were performed as per manufacturer's instructions, with the exception of Perkin Elmer reagents for chemiluminescent signal detection.

Ligand protein array

Differential expression of ERBB ligands was assessed using R&D Systems' Angiogenesis Array (ARY007), which probes for the following ERBB ligands: amphiregulin (AREG), epidermal growth factor (EGF), heparin-binding EGF (HB-EGF), and neuregulin β1/heregulin (NRGβ1/HRG). Cells were plated evenly into 6-cm dishes in serum-containing media. 24 hr later, the media was changed to a reduced volume of serum-free media for a period of 48 hr, after which the media was harvested and centrifuged to pellet cellular debris. 1 mL of the conditioned, serum-free media from each sample was added to the prepared protein array membranes. All remaining steps were performed as per manufacturer's instructions, except for the substitution of Perkin Elmer chemiluminescent signal detection reagents.

HER3 siRNA knockdown

Cells were plated evenly into 6-cm dishes in serum-containing media. The following day, cells were transfected using Lipofectamine RNAiMAX (Invitrogen / Life Technologies) as per manufacturer's instructions with 30 pmol final concentration of scrambled pool or siRNA against HER3. 24 hr after transfection, cells were treated with DMSO or 50 nM trametinib for another 24 hr. Cells were harvested for immunoblotting as described in the main methods section.

Integrated mutation profiling of actionable cancer targets (IMPACT) assay

DNA was extracted from cell lines using the Qiagen DNeAsy Tissue Kit or standard phenol-chloroform method. The samples were assayed for mutations and copy number alterations in 341 cancer-related genes as described previously [1]. Because cell lines and not patient tissues were genotyped, matched normal samples for germline SNP filtering were not available. Well-documented SNPs present at >1% allelic frequency were removed from the final report, however, rare germline SNPs may have been reported. Of note, a previously reported truncating mutation of NF1 (Q1336*) in the MeWo cell line was not detected in our analyses [2]. Results are reported in Supplementary Table S6, alongside the Vanderbilt Cancer Panel results.

Vanderbilt cancer panel for MiSeq

(See also, [3]). From literature review and mutation database research (Catalogue of Somatic Mutations in Cancer), we identified 66 genes that are mutated at a rate of greater than 1% in a majority of solid and liquid tumors (Supplementary Table S5). We instructed Illumina's online Design Studio program to design amplicon probes for use with the Illumina MiSeq platform against all exons of the 66 genes allowing an extension of 25 bases into the introns on either side of each exon. We also instructed the program not to avoid designing amplicon probes

around common single-nucleotide polymorphic (SNP) regions, because some somatic SNPs are often included in genomic SNP databases (example, EGFR c.2369C > T, p.T790 M in lung cancers resistant to first-line EGFR therapy) but are still important for biological function. Only 1536 amplicons are allowed per MiSeq probe panel; therefore, we split the genes into two capture panels (Supplementary Table S5). The number of target exons, cumulative target base-pairs, total number of amplicons per panel and the base-pair gap amount (i.e. base-pairs that were targeted but deemed unsuitable for an amplicon location) are listed in Supplementary Table S5 as well. Overall, the Illumina Design Studio was able to design probes against 95% and 93% of the intended exon targets in VCP1 and VCP2, respectively. Following sequencing, samples were analyzed for single-nucleotide variants (SNVs), and small insertions and deletions (indels) as follows: The Burrows-Wheeler Aligner (BWA) [4] was used to align sequencing reads to the UCSC human reference genome hg19. After alignment, SAMtools [5] was used to convert the alignment files to a sorted, indexed binary alignment map (BAM) format. To obtain the best sequence alignment for mutation-calling purposes, the BAM files were realigned using the Genome Analysis Toolkit (GATK) software [6]. Following reaglinment, the HaplotypeCaller program in GATK was used in its default setting to detect SNVs and indels from the BAM files. Finally, low quality variants were filtered out using VariantFiltration in GATK. Only high-confidence SNPs and indels were kept for downstream analysis. Results are reported concurrently with IMPACT assay results in Supplementary Table S5.

REFERENCES

1. Cheng DT, Mitchell T, Zehir A, Shah RH, Benayed R, Syed A, Chandramohan R, Liu ZY, Won HH, Scott SN, Brannon AR, O'Reilly C, Sadowska J, Casanova J, Yannes A, Hechtman J, et al. MSK-IMPACT: A Hybridization Capture-Based Next-Generation Sequencing Clinical Assay for Solid Tumor Molecular Oncology. J Mol Diagn. 2015.
2. Nissan MH, Pratilas CA, Jones AM, Ramirez R, Won H, Liu C, Tiwari S, Kong L, Hanrahan AJ, Yao Z, Merghoub T, Ribas A, Chapman PB, Yaeger R, Taylor BS, Schultz N, et al. Loss of NF1 in cutaneous melanoma is associated with RAS activation and MEK dependence. Cancer research. 2014; 74:2340–2350.
3. Meador CB, Jin H, de Stanchina E, Nebhan CA, Pirazzoli V, Wang L, Lu P, Vuong H, Hutchinson KE, Jia P, Chen X, Eisenberg R, Ladanyi M, Politi K, Zhao Z, Lovly CM, et al. Optimizing the Sequence of Anti-EGFR-Targeted Therapy in EGFR-Mutant Lung Cancer. Molecular cancer therapeutics. 2014.
4. Li H, Durbin R. Fast and accurate short read alignment with Burrows-Wheeler transform. Bioinformatics. 2009; 25:1754–1760.
5. Li H, Handsaker B, Wysoker A, Fennell T, Ruan J, Homer N, Marth G, Abecasis G, Durbin R. The Sequence Alignment/Map format and SAMtools. Bioinformatics. 2009; 25:2078–2079.
6. McKenna A, Hanna M, Banks E, Sivachenko A, Cibulskis K, Kernytsky A, Garimella K, Altshuler D, Gabriel S, Daly M, DePristo MA. The Genome Analysis Toolkit: a MapReduce framework for analyzing next-generation DNA sequencing data. Genome Res. 2010; 20:1297–1303.

SUPPLEMENTARY FIGURES AND TABLES

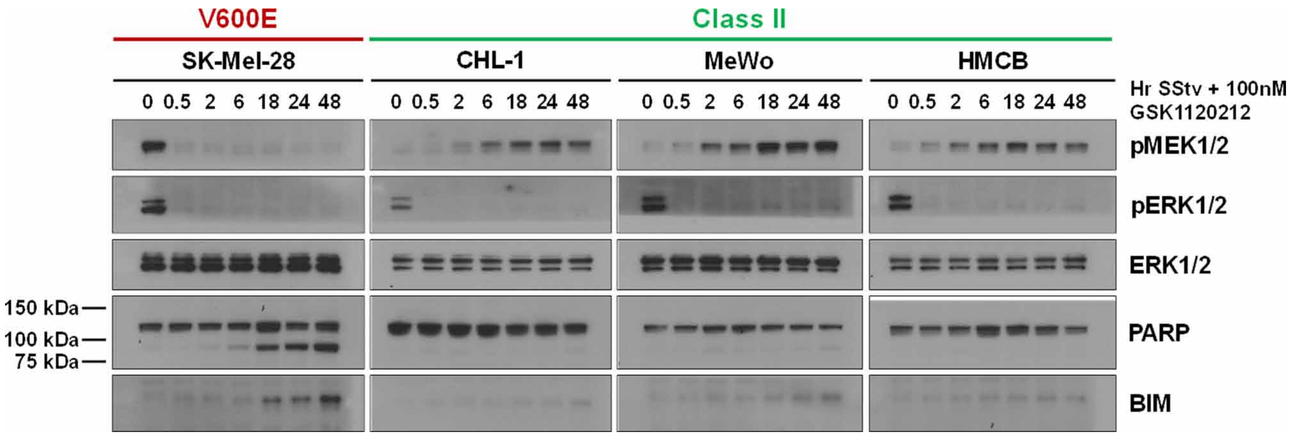

Supplementary Figure S1: Paradoxical activation of MEK1/2 in Class II pan-negative melanomas is sustained for at least 48 hours. Additionally, Class II melanomas display less cleaved PARP and less induction of pro-apoptotic Bim than the BRAF V600E-mutant line SK-Mel-28. GSK1120212 = trametinib; Hr SStv, hours of serum-starvation; nM, nanomolar; kDa, kilodaltons; p, phosphorylated.

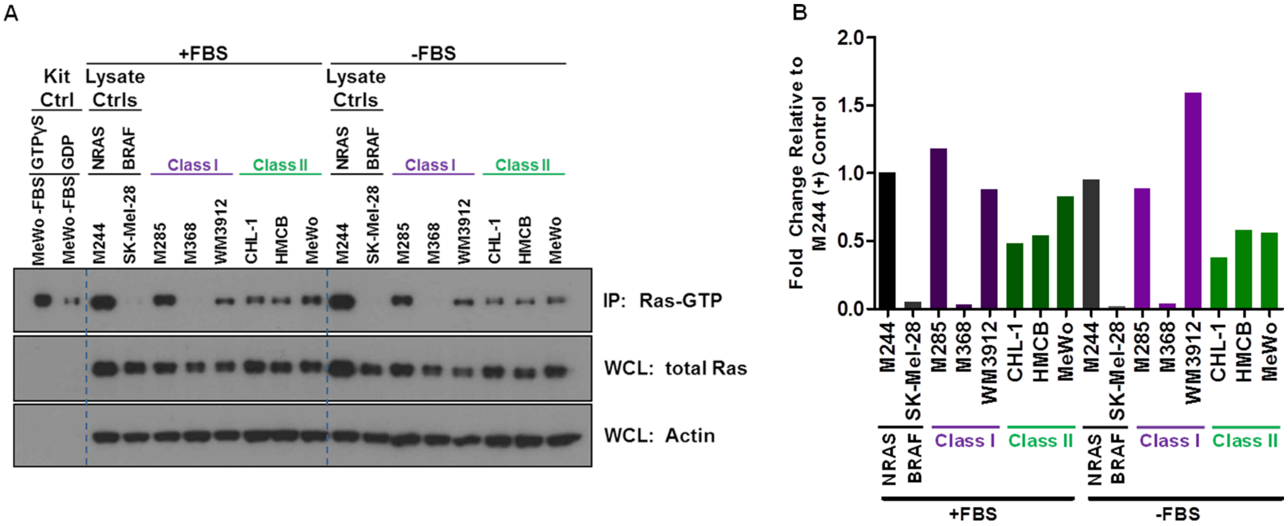

Supplementary Figure S2: No significant differences were observed in Ras-GTP (active Ras) between Class I and II cell lines. A. Immunoblot of the Ras-GTP pulldown results. **B.** Quantification of immunoblot results using ImageJ. Overall, both Class I and II pan-negative cell lines display lower Ras activity than an NRAS-mutant cell line. Ctrl(s), control(s); IP, immunoprecipitation; WCL, whole-cell lysate.

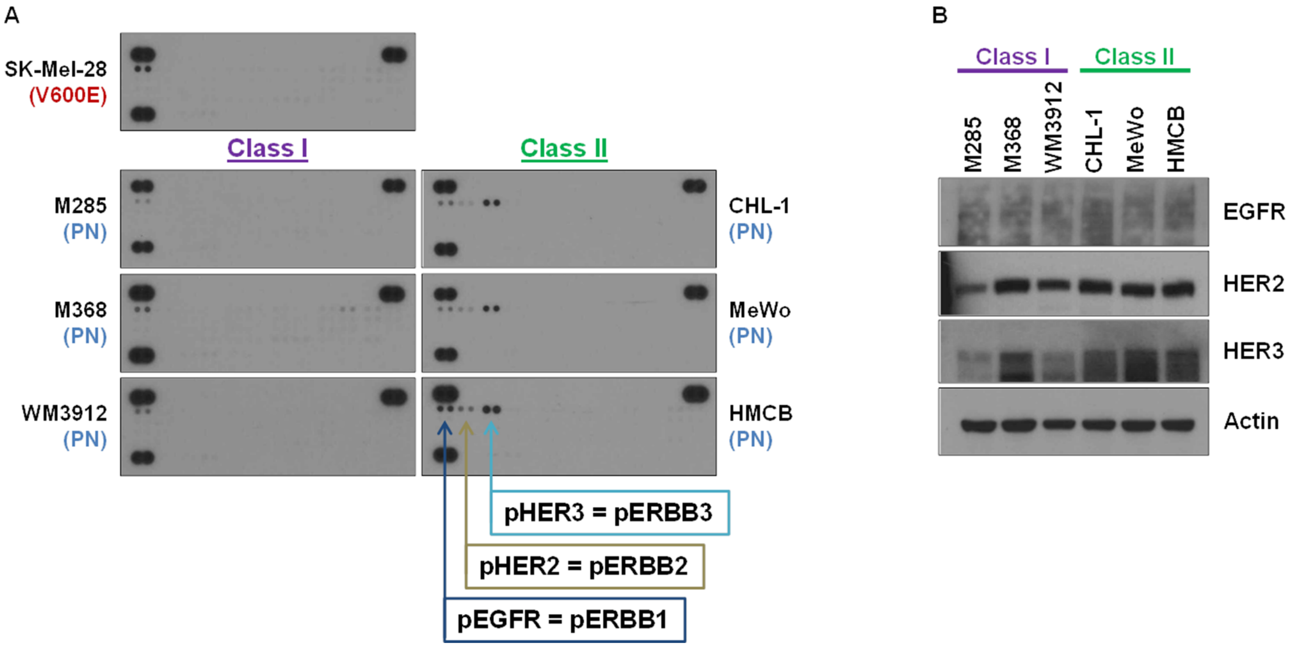

Supplementary Figure S3: Class II Pan-Negative Melanomas Display Increased Levels of Phosphorylated ERBBs 1, 2, and 3. A. Analysis of phosphorylated receptor tyrosine kinase arrays comparing V600-mutant, Class and Class II pan-negative melanomas reveals increased activity of EGFR, HER2 and HER3 in Class II lines. **B.** No significant differences were observed in total levels of EGFR, HER2, or HER3 between Class I and II lines.

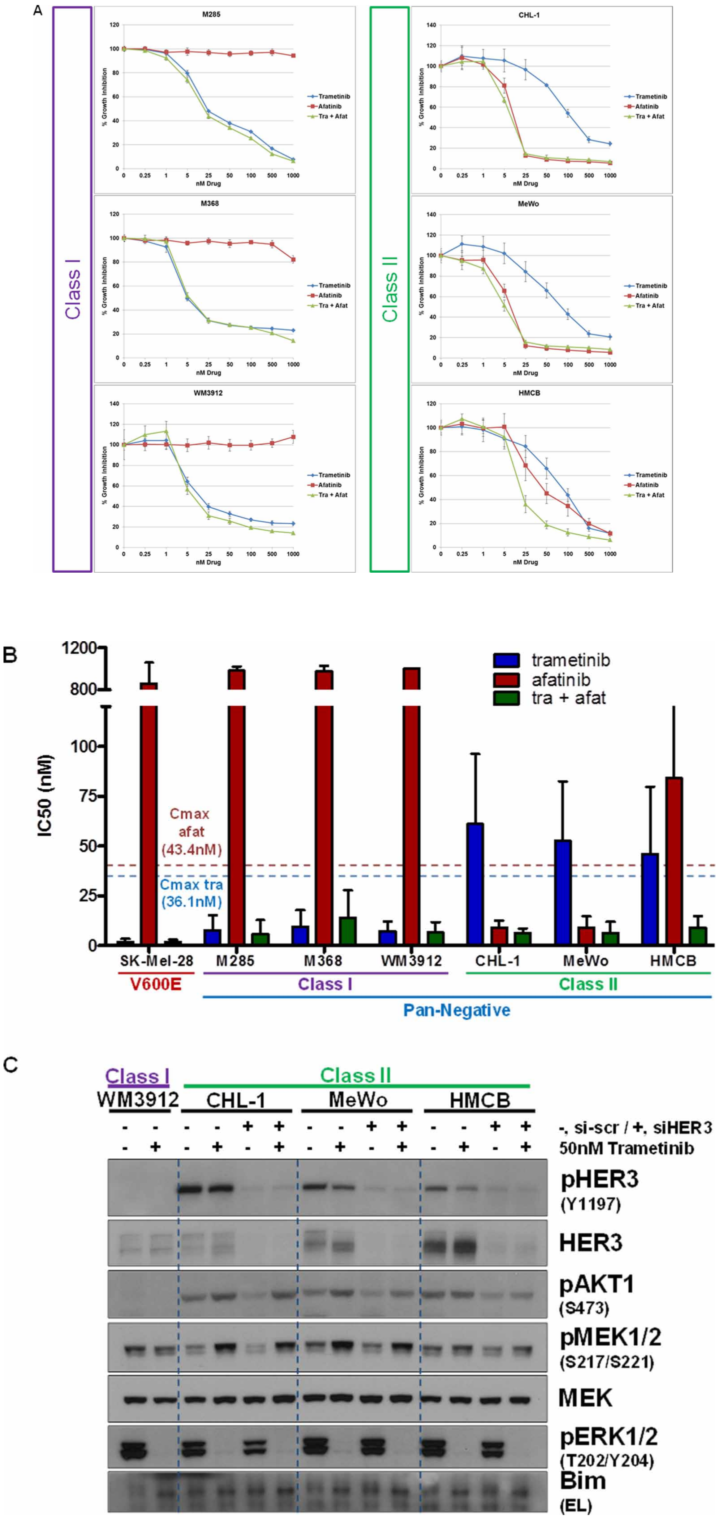

Supplementary Figure S4: Class II Pan-Negative Cell Lines are Sensitive to ERBB Inhibition. A. Class I and II lines were treated with increasing doses of trametinib, afatinib or the combination as described in the methods and assessed for cell viability. Class I lines are most sensitive to trametinib (Figure 1), but unlike Class II lines, are resistant to afatinib. **B.** Graph of IC50's derived from cell viability assays in (A). **C.** Immunoblot analysis of signaling following siRNA knockdown of HER3 in Class II lines reveals that although HER3 knockdown can attenuate AKT activation, it is less effective than small-molecule ERBB inhibition with afatinib (Figure 2C) and is less effective against ERK1/2 activation. nM, nanomolar; p, phosphorylated; si-scr, scrambled control siRNA; siHER3, HER3 siRNA.

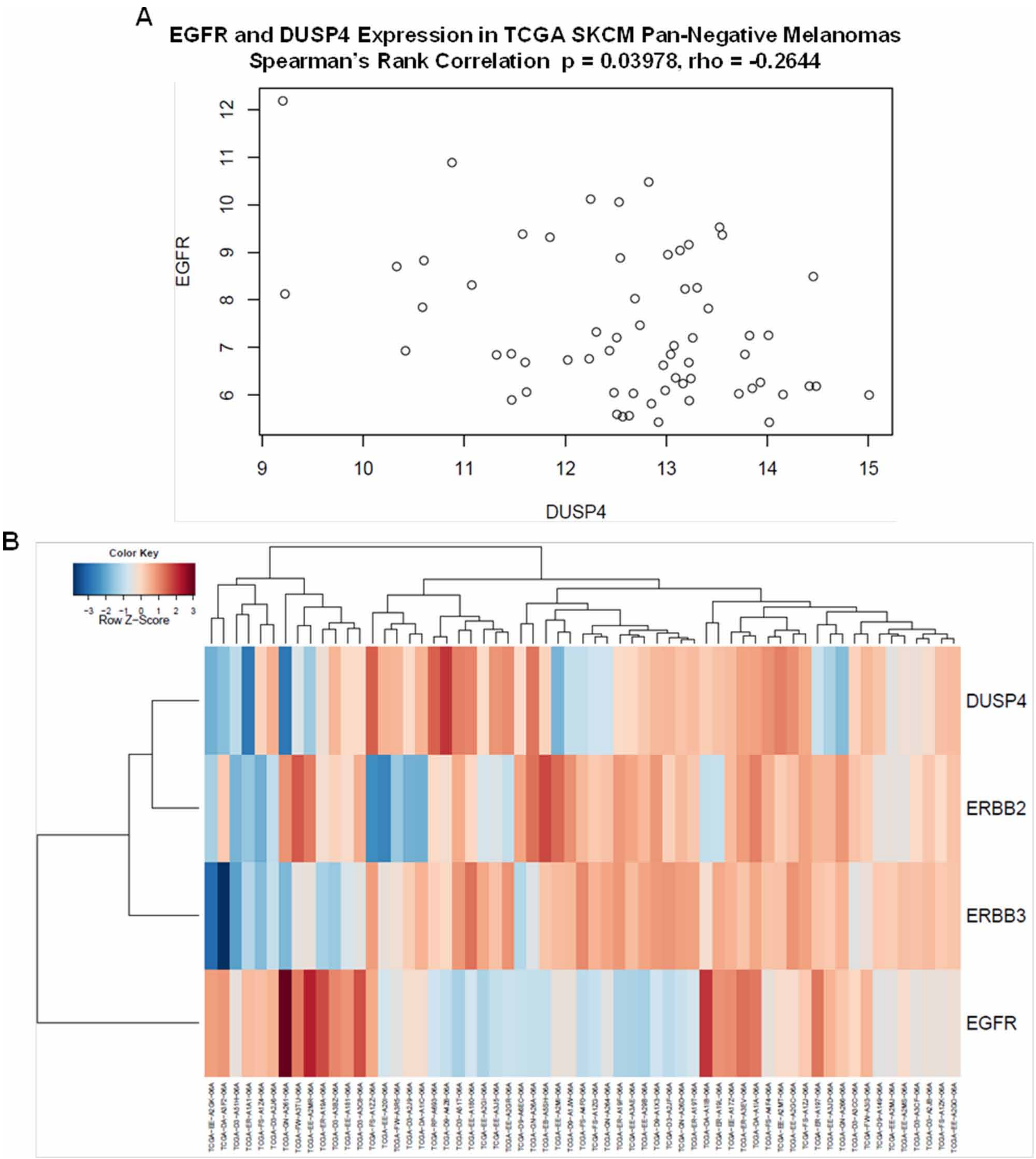

Supplementary Figure S5: Analysis of 61 Pan-Negative, Metastatic Melanomas in The Cancer Genome Atlas Reveals an Inverse Relationship between DUSP4 and EGFR expression. A. Based on expression values from RNA sequencing analysis, the Spearman's Rank Correlation was calculated for the expression of EGFR compared to DUSP4 in 61 pan-negative melanomas. These data reveal a statistically significant ($p = 0.03978$) inverse relationship between DUSP4 RNA expression and EGFR RNA expression, where rho (slope) = –0.2644. **B.** Heat map of expression levels of DUSP4, HER2/ERBB2, HER3/ERBB3 and EGFR across the 61 pan-negative melanomas again shows an inverse relationship between EGFR and DUSP4 expression.

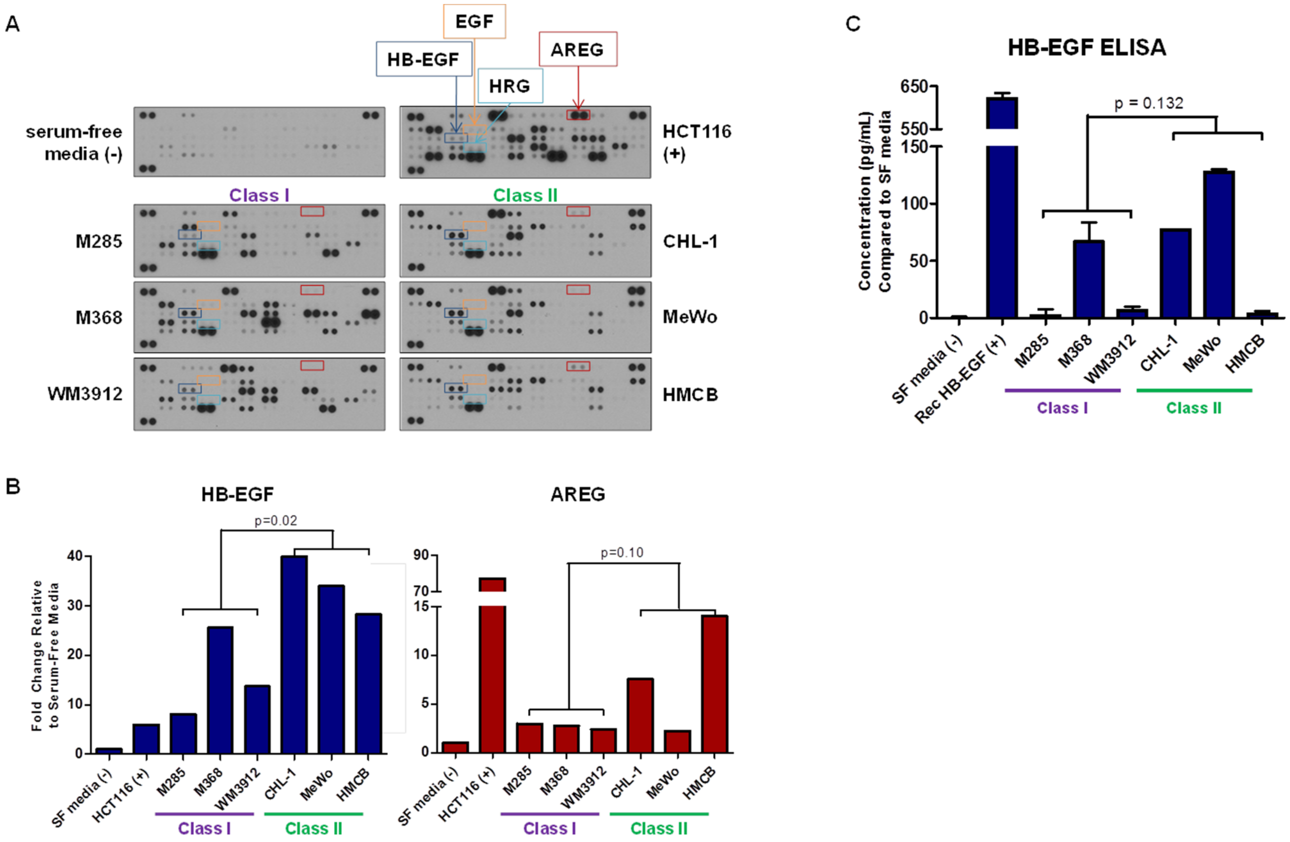

Supplementary Figure S6: Amphiregulin Expression is Higher in Class II Pan-Negative Melanomas Compared with Class I's. A. Protein array analysis of the expression of HB-EGF, EGF, HRG/NRGβ1, and AREG in serum-free conditioned media from HCT116 (positive control), Class I and Class II cell lines reveals that HB-EGF and AREG may be expressed at higher levels in Class II cells as quantified by ImageJ in **B. C.** ELISA for HB-EGF confirms a potential trend toward higher HB-EGF expression Class II cells versus Class I cells, however, it is not statistically significant. Refer to Figure 4D for AREG ELISA. SF media, serum-free media; EGF, epidermal growth factor; HB-EGF, heparin-binding EGF; AREG, amphiregulin; HRG, heregulin a.k.a. neuregulin-β1.

Supplementary Table S1: Alterations Assessed by the Vanderbilt Melanoma SNaPshot Assay

**BRAF**	**NRAS**
BRAF p.L597Q; c.1790T > A	NRAS p.G12C; c.34G > T
BRAF p.L597R; c.1790T > G	NRAS p.G12S; c.34G > A
BRAF p.L597S; c.1789_1790CT > TC	NRAS p.G12R; c.34G > C
BRAF p.V600R; c.1798_1799GT > AG	NRAS p.G12V; c.35G > T
BRAF p.V600K; c.1798_1799GT > AA	NRAS p.G12A; c.35G > C
BRAF p.V600E; c.1799T > A	NRAS p.G12D; c.35G > A
BRAF p.V600E; c.1799_1800TG > AA	NRAS p.G13A; c.38G > C
BRAF p.V600M; c.1798G > A	NRAS p.G13V; c.38G > T
BRAF p.V600G; c.1799T > G	NRAS p.G13R; c.37G > T
BRAF p.V600D; c.1799_1800TG > AT	NRAS p.G13D; c.38G > A
BRAF p.K601E; c.1801A > G	NRAS p.Q61E; c.181C > G
	NRAS p.Q61H; c.183A > T
**KIT**	NRAS p.Q61H; c.183A > C
KIT p.W557R; c.1669T > C	NRAS p.Q61L; c.182A > T
KIT p.W557R; c.1669T > A	NRAS p.Q61L; c.182_183AA > TG
KIT p.V559A; c.1676T > C	NRAS p.Q61K; c.181C > A
KIT p.V559D; c.1676T > A	NRAS p.Q61P; c.182A > C
KIT p.L576P; c.1727T > C	NRAS p.Q61R; c.182A > G
KIT p.K642E; c.1924A > G	NRAS p.Q61R; c.182_183AA > GG
KIT p.D816H; c.2446G > C	
	**GNA11**
**GNAQ**	GNA11 p.Q209P; c.626A > C
GNAQ p.Q209P; c.626A > C	GNA11 p.Q209L; c.626A > T
GNAQ p.Q209L; c.626A > T	
GNAQ p.Q209R; c.626A > G	

Certain melanoma-related CTNNB1 mutations are detected by this assay, however, they are not considered targetable at this time, and thus, will not be discussed in this article.

Supplementary Table S2: Results of Vanderbilt Melanoma SNaPshot Assay

Supplementary Table S3: Panel of 16 SNaPshot Pan-Negative Melanoma Cell Lines

Cell Line	Source	Culture Conditions
CHL-1	ATCC	DMEM + 10% FBS + 1% pen/strep
D35	QIMR (N. Hayward / C. Schmidt)	RPMI-1640 + 10% FBS + 1% pen/strep
HMCB	ATCC	DMEM + 10% FBS + 1% pen/strep
M285<br>M368<br>M375	UCLA (T. Ribas)	RPMI-1640 + 20% FBS + 1% pen/strep
MeWo	ATCC	DMEM + 10% FBS + 1% pen/strep
MM329	QIMR (N. Hayward / C. Schmidt)	RPMI-1640 + 10% FBS + 1% pen/strep
VP-Mel-20<br>VP-Mel-21<br>VP-Mel-36	VUMC	RPMI-1640 + 20% FBS + 1% pen/strep
WM3681<br>WM3912<br>WM3918<br>WM3928 / WM3928F<br>WM8 / WM1382	Wistar Institute (M. Herlyn)	4:1 MCDB-153 and Leibovitz's L-15 + 2% FBS + 1% pen/strep + 5 ug/mL bovine insulin + 1.68 mM calcium chloride

ATCC, American Type Cell Culture
QIMR, Queensland Institute of Medical Research
UCLA, University of California, Los Angeles
VUMC, Vanderbilt University Medical Center
MCDB-153 media, Sigma #M7403
Leibovitz's L-15 media, Gibco/Life Technologies, #11415–064

Supplementary Table S4: Previous Studies Citing Observation of Paradoxical MEK Activation in Response to MEK Inhibition

		MEK and ERK Response by Genotype				
**Reference**	**MEK Inhibitor**		**BRAF V600**	**RAS-mutant**	**WT**	**Cited Mechanism of Paradoxical MEK Activation**
Ishii, *Cancer Res*, 2013	PD-0325901	pMEK<br>pERK	↔<br>↓	↑<br>↓	↔<br>↓	RAS mutation
Emery, *PNAS*, 2009	AZD6244	pMEK<br>pERK	↔↑<br>↓	n/a<br>n/a	n/a<br>n/a	MEK1 mutation (AZD6244-resistant line derived from a patient)
Villanueva, *Cell Reports*, 2013	Trametinib or AZD6244	pMEK<br>pERK	↔↑<br>↓	n/a<br>n/a	n/a<br>n/a	Concurrent BRAF amplification and MEK2 mutation (also confers resistance to RAFi)
Wagle, *Cancer Discovery*, 2014	Trametinib	pMEK<br>pERK	↔↑<br>↓	n/a<br>n/a	n/a<br>n/a	MEK2 mutation
von Euw, *Molec Cancer*, 2012	TAK-733	pMEK<br>pERK	↔<br>↓	↑<br>↓	n/a<br>n/a	RAS mutation Also tested GNAQ/11-mut lines: same paradox
Pratilas, *PNAS*, 2009	PD-0325901	pMEK<br>pERK	↔↓<br>↓	n/a<br>n/a	↑<br>↓	WT cells are driven by RTK signaling

Cells highlighted in pink indicate instances of paradoxical MEK1/2 activation (↑) upon MEK1/2 inhibition. WT, wild-type.

Supplementary Table S5: Vanderbilt Cancer Panel (VCP) Design Summary[a]

VCP1			VCP2		
Number of Target Exons = 594			Number of Target Exons = 457		
Cumulative Target bp = 195838 bp			Cumulative Target bp = 210570 bp		
**# Amplicons = 1494 (max 1536)**			**# Amplicons = 1448 (max 1536)**		
# Gaps = 80			# Gaps = 123		
Total Gap Distance = 9734 bp			Total Gap Distance = 15361 bp		
Amplicon Coverage = 95%			Amplicon Coverage = 93%		
Low-Scoring Targets = 13			Low-Scoring Targets = 13		
**Gene**	**Chr**	**#Exons**	**Gene**	**Chr**	**#Exons**
AKT1	14	16	AKT2	19	14
ALK	2	29	AKT3	1	15
BRAF	7	18	ARAF	X	16
CDK4	12	8	BCL2	18	4
DDR2	1	19	BCL2L1	20	4
EGFR	7	31	ERBB3	12	29
ERBB2	17	31	ERBB4	2	28
FGFR1	8	23	FGFR4	5	20
FGFR2	10	24	HRAS	11	8
FGFR3	4	19	JAK1	1	25
GNA11	19	7	JAK2	9	25
GNAQ	9	7	JAK3	19	24
IDH1	2	10	KDR	4	30
IDH2	15	11	MCL1	1	5
KIT	4	22	MYC	8	3
KRAS	12	6	MYCL1	1	5
MAP2K1	15	11	MYCN	2	3
MAP2K2	19	11	NOTCH1	19	33
MET	7	22	NOTCH2	1	35
MLH1	3	22	NOTCH3	9	34
MLH3	14	13	NTRK1	1	19
MSH2	2	16	NTRK2	9	24
MTOR	1	58	NTRK3	15	21
NF1	17	59	PTCH1	9	30
NF2	22	20	PTCH2	1	24
NRAS	1	7	RAF1	3	17
PDGFRA	4	23	RB1	13	27
PIK3CA	3	21	RET	10	21

(*Continued*)

VCP1			VCP2		
PTEN	10	9	SMAD4	18	12
RICTOR	5	38	STK11	19	10
RPTOR	17	34	TP53	17	15
SMO	7	12	IGF1R	15	21
TSC1	9	24			
TSC2	16	43			
**Total Genes = 34**			**Total Genes = 32**		

See also, Meador, et al., Molec Cancer Therapeutics, 2014 (PMID: 25477325)

Supplementary Table S6: Results of MSKCC IMPACT and Vanderbilt Cancer Panel Mutational Analyses in Genes with Known Significance in Melanoma and Additional Genes Relevant to the MAPK and PI3K/AKT Pathways
